# Molecular identification of *Babesia canis canis* genotype A in a dog from Iran

**DOI:** 10.1002/vms3.630

**Published:** 2021-09-12

**Authors:** Milad Ghasemzade, Bijan Esmaeilnejad, Siamak Asri‐Rezaei, Mojtaba Hadian

**Affiliations:** ^1^ Department of Pathobiology, Faculty of Veterinary Medicine Urmia University Urmia Iran; ^2^ Department of Internal Medicine and Clinical Pathology Faculty of Veterinary Medicine, Urmia University Urmia Iran

**Keywords:** 18S rRNA, *Babesia canis canis*, dog, genotype A

## Abstract

**Background:**

Canine babesiosis is a common and clinically significant tick‐borne disease caused by obligate haematozoan parasites of the genus Babesia.

**Purpose:**

To report Babesia canis canis genotype A infection in a dog.

**Methods:**

A 2‐year‐old female Shih Tzu dog was submitted with the history of anorexia and depression for one week and no prior surgery. Fever, anorexia, depression and vomiting as well as mucosal pallor were noticed on physical examination. Microscopic examination of the Giemsa‐stained blood smear disclosed large form of Babesia, and single to four pear‐shaped merozoites within erythrocytes (RBCs). The specific primers were used for detecting Babesia canis.

**Results:**

The result of PCR was confirmed by 18S rRNA gene sequence analyzing and has been registered in GenBank under following accession numbers for Babesia canis canis (MW199108). The sequences were compared to those in GenBank, and alignments showed that the B. canis canis isolate belonged to genotype A.

**Conclusions:**

This is the first description of B. canis canis genotype A in dog from Iran.

## Abstract 2

A dog infected by B. c. canis genotype A. This is the first description of B. canis canis genotype A in dog from Iran.

## 1 INTRODUCTION

Canine babesiosis is a common and clinically significant tick‐borne disease caused by obligate haematozoan parasites of the genus *Babesia*. Lethargy, anorexia, fever, jaundice, haemolytic anaemia, haemoglobinuria/bilirubinuria, weight loss and sometimes death are the most common encountered clinical signs of the acute infection (Uilenberg, 2006). Detection of the pathogen in Giemsa‐stained peripheral blood samples under a light microscope has long been established for the diagnosis of *Babesia* and other haematozoans. However, this morphology‐based approach is labour and time consuming because of its low sensitivity. Particularly, the efficiency of the method is undermined in cases of mixed infection or low parasitaemia which results in a false negative diagnosis (Oyamada et al., 2005). To circumvent these pitfalls, novel methods including molecular assays, such as polymerase chain reaction (PCR) have been developed. PCR and sequence analysis offer a remarkably higher sensitivity and specificity enabling the differentiation of haematozoan parasites (Shabani et al., 2020).

Based on morphology, the causative agents of canine babesiosis have been divided into two distinct groups, namely the large *Babesia* measuring 3–5 μm *B. canis* which mainly appears to be pyriform, and the smaller counterpart with the size of 1–3 μm, known as *B. gibsoni* which mainly appears to be signet ring form (Laha et al., 2015). Molecular analysis has demonstrated three distinct subspecies of *Babesia canis* including *B. canis rossi*, *B. canis canis* and *B. canis vogeli* (Solano‐Gallego et al., 2016; Wang et al., 2019). These subspecies are morphologically similar but have different vectors and pathogenicity which are now considered to be separate species (Solano‐Gallego et al., 2016). Moreover, an unnamed *Babesia* species that is closely related to *Babesia bigemina* has been reported in North Carolina in the United States (Boozer & Macintire, 2003). Three small *Babesia* species are known to infect canines including *B. gibsoni, B. conradae* and *B. microti*‐like spp (Zahler et al., 2000).

*B. canis canis* infection is distributed in Europe and Asia, which is a moderate virulent‐subspecies, potentially causing a wide range of clinical signs in dogs (Boozer & Macintire, 2003). *Rhipicephalus sanguineus* has been implicated in the transmission of this parasite in Iran. Little is known about molecular identification of *B. canis* spp in Iran (Habibi et al., 2020). The present study presents the first description of molecular and phylogenetic analysis of *B. canis* subspecies *canis* genotype A in blood sample collected from a dog.

## 2 CASE HISTORY

In September 2020, a 2‐year‐old female Shih Tzu dog weighing 12 kg was examined by a veterinary clinician in Tehran (35.6892′ N, 51.3890′ E), capital city of Iran. She had not travelled abroad. The owner reported anorexia, depression and vomiting without history of prior surgery. Pale mucous membrane, mild dehydration, 40˚C body temperature, 135 beat/min heart rate and 23 breath/min respiratory rate were recorded on physical examination. Blood sample from the saphenous vein was collected in EDTA tubes (Kendall Co., Mansfield, USA) for molecular analysis. Giemsa‐stained blood smear was examined for the presence of the canine piroplasms and other haemopathogens (Furlanello et al., 2005). Then, based on the results obtained from the microscopic examination, molecular assays were performed as below.

The DNA was extracted from blood using a DNA extraction kit (MBST, Tehran, Iran). Specific PCR assay for canine babesiosis was performed according to the previously described method for detection and differentiation of three *B. canis* subspecies and *B. gibsoni* based on 18S rRNA gene sequence by semi‐nested PCR technique. For the PCR, an outer primer pair (455‐479F and 793‐772R) was designed which would amplify an approximately 340 bp fragment from *B. canis* subspecies *canis* that spanned a hypervariable region of the 18S rRNA gene. Then, specific internal primer was designed for *B. canis* subspecies *canis* (BCC‐F) that was paired with the outer reverse primer in the semi‐nested secondary reaction to amplify 198 bp fragment (Birkenheuer et al., 2003). The PCR assay was conducted in a final reaction volume of 20 μl containing 10X PCR premixed (Cinnagen, Tehran, Iran), 6 μl ddH2O, 10 pmol of each primer and 2 μl of DNA template using an automatic DNA thermal cycler (CP2‐003; Corbett Research, Sydney, Australia) with the first denaturation at 94˚C for 3 min followed by 35 cycles consisting of a denaturing step of 10 s at 94˚C, an annealing step of 20 s at 58˚C and an extension step of 35 s at 72˚C. Finally, the reaction was terminated with an extension step of 5 min at 72˚C. Semi‐nested PCR (BCC‐F and 793‐772R) was performed in separate tubes under the same conditions, except for the number of cycles. The PCR product was electrophoresed in 1.5% (w/v) agarose gel (containing 7 μg ethidium bromide in 0.5X TBE electrophoresis buffer) for 1 h at 75 V and visualized under a UV trans‐illuminator (Synoptics Ltd., Cambridge, UK). DNA samples from known 18S rRNA *Babesia* positive case (MH793502) and distilled water sample were used as positive and negative controls, respectively. The secondary PCR products were purified from the agarose gel using purification kit (Bioneer, Daedeok, South Korea) according to the manufacturer's instructions. An amount of 15 μl of purified PCR product and 10 μl of each of forward and reverse primers (5 μM) were sent for sequencing (SinaClon, Tehran, Iran). The obtained 18S rRNA sequence was compared to GenBank entries using the BLAST tool provided by National Center for Biotechnology Information (NCBI).

For the classification of the sequences, the nucleotide sequence data was separately aligned against different related dog *Babesia* sequences existing in GenBank by MEGA 7.0. Creation of multiple‐sequence alignment was established using Clustal W programme in the MEGA 7.0 software for each query DNA sequence. Data sequences were also used for construction of the phylogenetic trees using maximum parsimony and neighbour‐jointed methods. All positions containing gaps and missing data were eliminated (Tamura et al., 2007). Furthermore, to assess *B. canis canis* genotypes, the sequences obtained in this study were compared with those of group A (AY703072) and group B (AY649326) (Adaszek et al., 2009; Hornok et al., 2016).

## 3 RESULTS AND DISCUSSION

**FIGURE 1:**
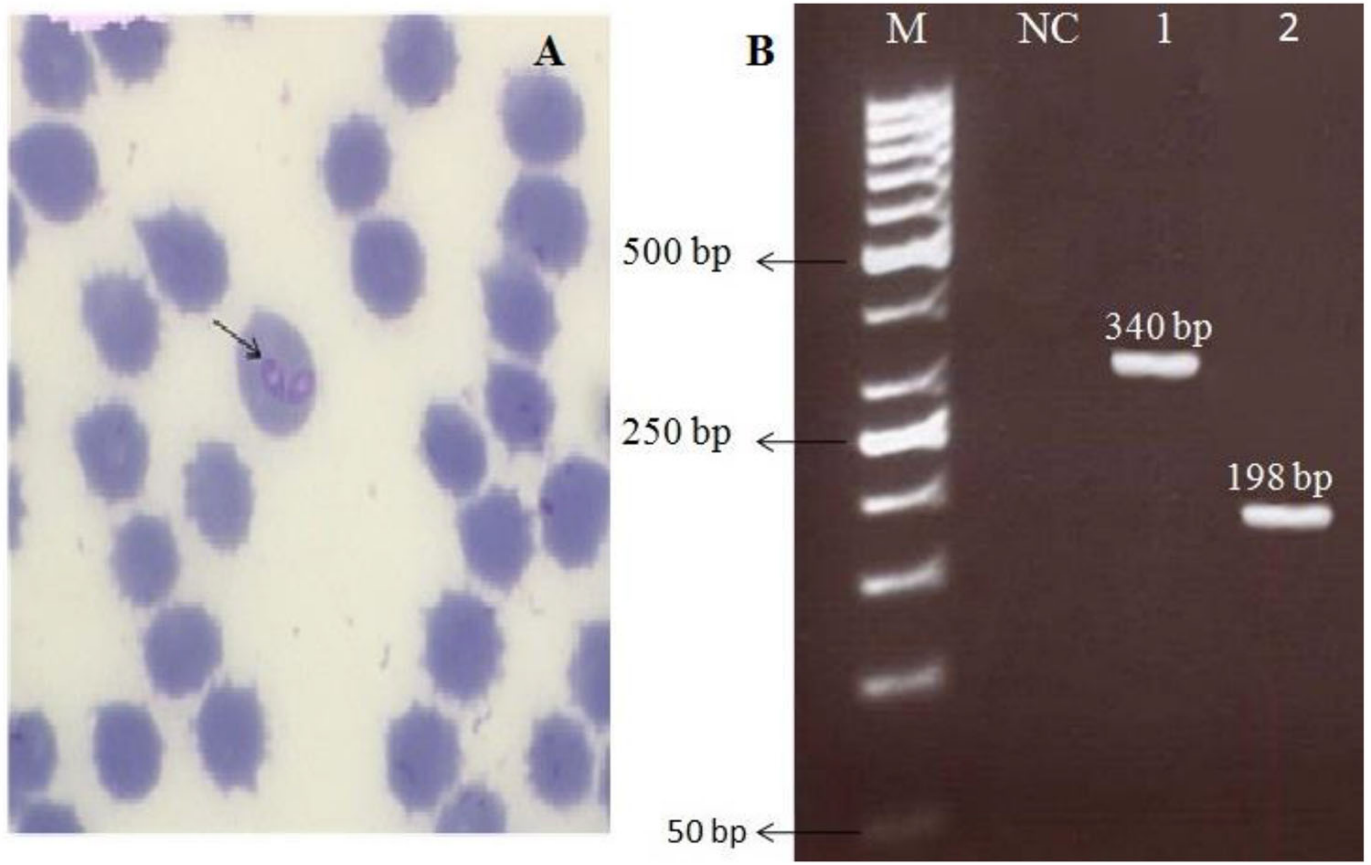
(a) Giemsa‐stained blood smear showing merozoites of large *Babesia* spp (100×). (b) Polymerase chain reaction (PCR) analysis with primers 455–479F and 793‐772R specific for 18S rRNA gene of *B. canis* (Lane 1), the corresponding PCR product from lane 1 was analyzed by seminested PCR using *B. canis* subspecies *canis* specific primers BCC‐F and 793‐772R (lane 2). Molecular weight marker (lane M: 50 bp) and negative (i.e., no‐DNA) control (NC)

Light microscopic examination revealed the presence of large form of *Babesia* which characteristically pointed one end and round other. Therefore, canine babesiosis due to a large form of the parasite, that is *B. canis*, was diagnosed. The molecular analysis confirmed infection with *B. canis canis* (an expected 340 bp and 198 bp). Meanwhile, no PCR amplification products were observed in negative control (Figure 1).

**FIGURE 2:**
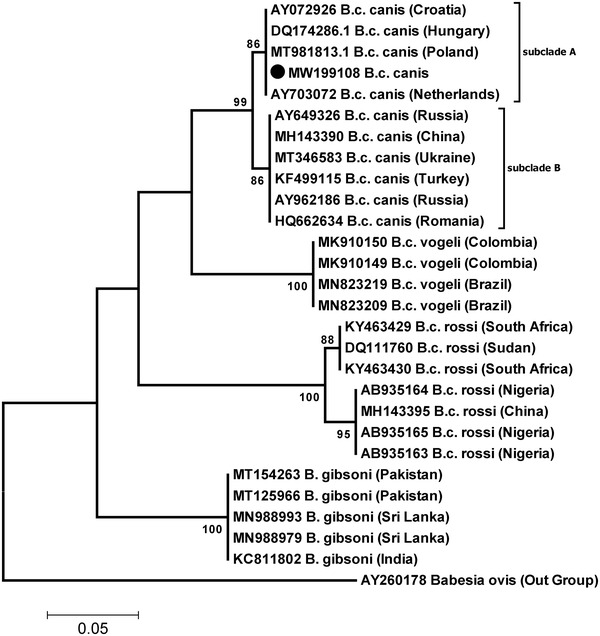
Phylogenetic tree inferred from the sequences of the *Babesia* spp. Molecular phylogenetic analysis of the Iranian isolate of *B. canis canis* (MW199108) obtained from dog in the present study and other related sequences from the GenBank based on the partial 18SrRNA sequences. The black circle indicates the newly generated sequence of this study. Bootstrap values obtained from 1000 replications are provided on each branch. Scale bar represents nucleotide substitutions per position

The sequence was designated as ‘Tehran isolate’ and submitted to the GenBank database with the accession number MW199108. Comparative sequence analysis using the obtained 18S rRNA sequence (MW199108: 198 bp) demonstrated the highest homology (100%) to previously registered *B. canis canis* sequences from Croatia, Hungary, Netherland and Poland. Moreover, the sequences were grouped in two major genotypes A and B (Figure 2).

**FIGURE 3:**
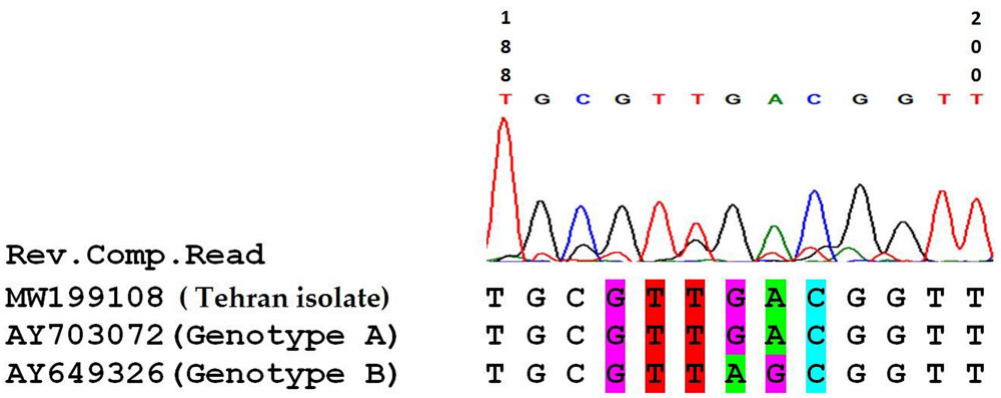
Comparison of a part of *B. canis canis* sequence (Tehran isolate) obtained in this study with those of genotypes A and B

Following the comparison of the obtained nucleotide sequence with those of genotypes A and B, the isolate shared 100% similarity with genotype A and the main difference was observed in positions 194 and 195. The differences of the two genotypes in the row of adenine and guanine nucleotides are in genotype A as GA and AG in genotype B (Figure 3). The parasitaemia was estimated to be 2.4%.

The first study reporting canine babesiosis in Iran was published in 1973. In that study, 155 dogs and one fox were included from the north of Iran. Eighty‐six dogs were splenectomized and the blood was examined at two‐day intervals. Blood smears from one splenectomized dog were found to contain *B. canis*. The fox was also identified to be infected with *B. gibsoni* (Niak et al., 1973). Since then, sporadic cases of this disease were reported from different regions of Iran. However, these studies could only characterize the parasite at species level (Akhtardanesh et al., 2016; Bigdeli & Namavari, 2017; Bigdeli et al., 2012; Razi et al., 2013). Just recently, Habibi et al. (2020) conducted a molecular study at subspecies level and detected *Babesia canis vogeli* in 10 dogs (out of 40) in Tehran. Therefore, this is the first description of molecular characterization and genetic diversity of *Babesia canis canis* isolated from Iran.

On the basis of 18S rRNA gene sequence analysis, genetic heterogeneity of *B. canis canis* has been documented in Poland, Netherland, Hungary and China. Two genotypes of *B. canis canis*, including genotypes A and B, have been described with different virulence (Adaszek et al., 2009). Based on clinical signs and mortality rates, the genotype B is considered to be more virulent than the genotype A (Wang et al., 2019). The clinical manifestations of *B. canis canis* infection are moderate to severe and include petechiae, epistaxis, vomiting and anaemia. The severity of the disease depends on the species of *Babesia* causing infection and other factors such as immune status and age of the host (Wang et al., 2019).

In the present study, the clinical signs of the infected dog were anorexia, depression, vomiting, fever and paled mucosa which were similar to those reported by Wang et al (2019). Although the clinical signs of the both studies were almost the same, the causative agent detected by Wang et al. (2019) was genotypes B. Several parameters such as age, breed and other than the causative agent may influence clinical manifestation of canine babesiosis. More importantly, the sample size was relatively small in this and the parasitaemia was high which potentially overshadow the clinical findings.

Genetic diversity plays a crucial role in the survival of piroplasm inside the hosts. Considering that molecular prevalence of canine babesiosis is as high as 18.2% in Iran, molecular characterization at subspecies levels would significantly contribute.

Our study was the first description of *B. canis canis* infection in a dog from Tehran, Iran. Our data provide valuable insights into the presence of the genotype A in the country. Considering that the molecular characterization at subspecies level has rarely been performed throughout the country, similar studies should urgently be conducted.

## CONFLICT OF INTEREST

The authors declare no conflict of interest.

## AUTHOR CONTRIBUTION

*Formal analysis, validation and writing‐review & editing*: Milad Ghasemzade. *Conceptualization, data curation, funding acquisition, investigation, methodology, project administration, supervision, validation, visualization, writing‐original draft and writing‐review & editing*: Bijan Esmaeilnejad. *Conceptualization, data curation, funding acquisition, investigation, methodology, project administration, supervision, validation, visualization and writing‐review & editing*: Siamak Asri‐Rezaei. *Formal analysis, investigation, resources, software and validation*: Mojtaba Hadian.

## ETHICS STATEMENT

The authors would like to thank the Office of the Vice Chancellor for Research of Urmia University for financial support of this study. This paper is part of DVM thesis of Milad Ghasemzade, numbered 3927, under supervision of Drs. Bijan Esmaeilnejad, Siamak Asri‐Rezaei.

## Data Availability

The data that support the findings of this study is openly available in GenBank at https://www.ncbi.nlm.nih.gov/nuccore/, reference number MW199108.
